# S16-4 SLOfit Lifelong: citizen science approach to promote and maintain physical fitness and physical literacy across the lifespan

**DOI:** 10.1093/eurpub/ckae114.273

**Published:** 2024-09-26

**Authors:** Colin O'Hehir, Paul Brosnan

**Affiliations:** Department of Health Ireland; Department of Health Ireland

## Abstract

**Purpose:**

SLOfit Lifelong is a public health initiative which was created to upgrade a well-established, national physical fitness surveillance system for Slovenian schoolchildren that has been collecting annual fitness data for over three decades.

**Project or policy description:**

The ultimate objective of creating SLOfit Lifelong was to build a modern societal infrastructure with the capacity and ability to detect future causal associations between childhood physical fitness trends and future health outcomes based on the lifelong surveillance of one's own fitness status. By instilling citizens with an ambition to test, understand, and follow-up their own physical fitness and health status (including related health risk factors), this initiative provides the technical support and expert feedback needed to engender greater individual control over understanding (and thus modulating), one's own physical fitness status as they progress into older adulthood.

**Conclusions:**

Through its sophisticated online web application My SLOfit, social media, print media, and outreach workshops, SLOfit Lifelong provides the expert support for public health engagement by fostering positive lifelong physical literacy experiences an individual can enjoy across their aging journey.

